# Doxorubicin Delivery Using pH and Redox Dual-Responsive Hollow Nanocapsules with a Cationic Electrostatic Barrier

**DOI:** 10.3390/pharmaceutics9010004

**Published:** 2016-12-30

**Authors:** Ryoma Teranishi, Ryota Matsuki, Eiji Yuba, Atsushi Harada, Kenji Kono

**Affiliations:** Department of Applied Chemistry, Graduate School of Engineering, Osaka Prefecture University, 1-1 Gakuen-cho, Naka-ku, Sakai, Osaka 599-8531, Japan; ss108056@edu.osakafu-u.ac.jp (R.T.); journey0215@gmail.com (R.M.); yuba@chem.osakafu-u.ac.jp (E.Y.); kono@chem.osakafu-u.ac.jp (K.K.)

**Keywords:** hollow nanocapsules, doxorubicin, dual responsive, electrostatic barrier, drug delivery system, self-assembled polymers

## Abstract

For the delivery of doxorubicin (DOX), pH and redox dual responsive hollow nanocapsules were prepared through the stabilization of polymer vesicles, which spontaneously formed from polyamidoamine dendron-poly(l-lysine) (PAMAM dendron-PLL), by the introduction of disulfide (SS) bonds between PLLs. The SS-bonded nanocapsules exhibited a very slow release of DOX under an extracellular environment because the cationic PLL membrane acted as an electrostatic barrier against the protonated DOX molecules. However, increasing the glutathione concentration to the intracellular level facilitated the immediate release of DOX through the collapse of nanocapsules by the spontaneous cleavage of SS bonds. SS-bonded nanocapsules also escaped from the endosome by the buffering effect of PAMAM dendrons, and DOX delivery into the cytoplasm was achieved. Furthermore, DOX molecules delivered by SS-bonded nanocapsules exhibited an effective in vitro anticancer effect to HeLa cells.

## 1. Introduction

Chemotherapy is a major treatment approach against cancer that has reduced patient mortality rates. However, direct administration of anticancer drugs has strict dose limitations because of the serious adverse side effects, resulting in low therapeutic effects [1]. As a promising method for cancer therapy, drug delivery systems (DDSs), including nanocarriers such as polymer micelles or vesicles, have succeeded in reducing side effects and improving the bioavailability of anticancer drugs [2,3,4,5,6,7]. DDS nanocarriers offer several distinct advantages for anticancer drugs, such as improved solubility and prolonged in vivo circulation times by the suppression of the immediate diffusion of drugs into normal tissues. These nanocarriers are required to have several features: (i) the construction of well-defined structures to entrap anticancer drugs stably; (ii) the ability for endosomal escape; and (iii) the release of the encapsulated drugs at their target sites in response to intracellular environmental changes. Polymer micelles and vesicles with intracellular environment-responsive behaviors (i.e., pH, enzyme, and reductive environment) are of special interest for DDS nanocarriers [8,9,10,11,12,13]. The uptake of most nanocarriers is via the endocytosis pathway. Here, nanocarriers pass through the endosome and lysosome under mild acidic conditions, and move to the cytoplasm with a reductive environment. For effective delivery of drug molecules into the cytoplasm, functional groups, e.g., tertiary amines and carboxylates, that can be protonate in response to a decrease in pH from physiological to endosomal or lysosomal pH for endosomal escape are introduced to nanocarriers. Additionally, a disulfide (SS) bond is often used as a cross link that responds to a reductive environment, and stable SS bonds under extracellular conditions can be cleaved in an intracellular reductive environment. The encapsulated drugs in nanocarriers can be promptly released through the cleavage of SS bonds in the cytoplasmic reductive condition.

**Scheme 1:**
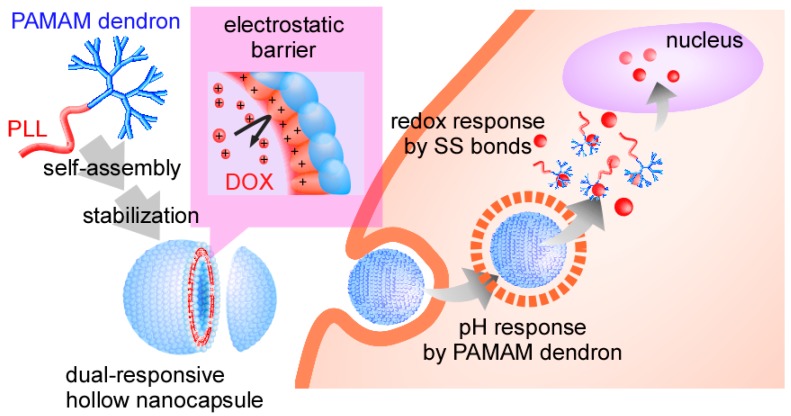
The concept of doxorubicin (DOX) delivery using pH and redox dual-responsive nanocapsules with an electrostatic barrier.

We have investigated self-assembled polymer vesicles of head-tail type polycations composed of a polyamidoamine dendron head and a poly(l-lysine) tail (PAMAM dendron-PLL) as a nanocarrier in the DDS field [14,15,16,17]. PAMAM dendron-PLL spontaneously forms polymer vesicles with a narrow size distribution through a coil-to-helix transition of PLL tails in a mixing solvent of water and methanol with high methanol content [18], and hollow nanocapsules were successfully prepared through the introduction of covalent or SS cross-linkages between primary amines in PLL tails in polymer vesicles [19,20]. The SS-bonded nanocapsules have pH and redox dual responses, in which nanocapsules respond to a decrease in pH from physiological to endosomal pH and an increase in glutathione levels. With both features, the nanocapsules can escape from the endosome and release the entrapped molecules through destabilization of the nanocapsules in the cytosol. Importantly, the protonated SS-bonded PLL membrane in the nanocapsule functions as an electrostatic barrier against the cationic molecules entering the nanocapsules, and the entrapped cationic molecules in the nanocapsules cannot be released from the nanocapsules due to the presence of this electrostatic barrier [20]. Such properties of SS-bonded nanocapsules may be suitable for the delivery of doxorubicin (DOX), because DOX has a primary amine with a p*K*_a_ of 8.3 [21,22] and is cationic at physiological pH (pH 7.4). In this study, we evaluated the delivery of DOX using pH and redox dual responsive nanocapsules that were prepared through the stabilization of PAMAM dendron-PLL polymer vesicles using SS bonds between PLLs (Scheme 1). This nanocapsule was capable of delivering DOX into the cytosol of HeLa cells, and the delivered DOX exhibited effective anticancer effects.

## 2. Materials and Methods

### 2.1. Materials 

**Scheme 2:**
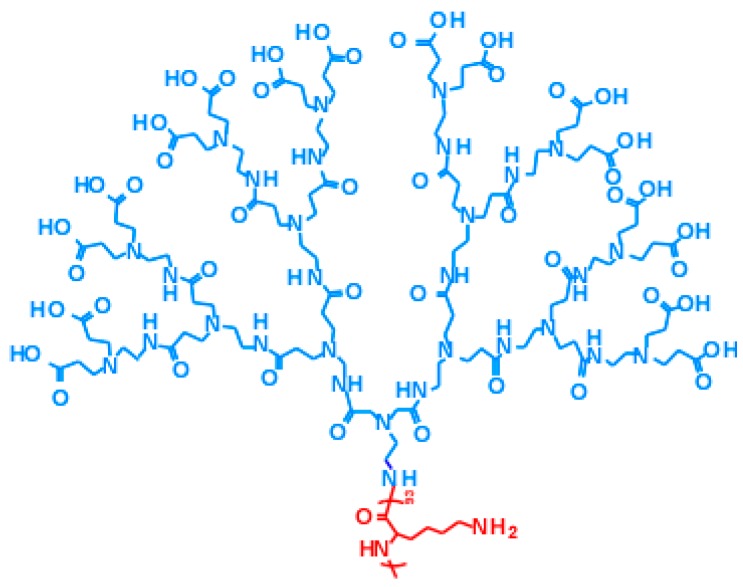
The chemical structure of polyamidoamine dendron-poly(l-lysine) (PAMAM dendron-PLL).

Polyamidoamine dendron-poly(l-lysine) block copolymer (PAMAM dendron-PLL), which has a PAMAM dendron head with a 3.5th generation and a PLL tail with a 93 polymerization degree, was synthesized according to a previous report [14,18]. The chemical structure of PAMAM dendron-PLL is shown in Scheme 2. 2-Iminothiolane hydrochloride (IT) was purchased from Sigma-Aldrich (St. Louis, MO, USA). Ethylene glycol diglycidyl ether (EGDE) and reduced glutathione (GSH) were purchased from Tokyo Chemical Industry Co. Ltd. (Tokyo, Japan). Doxorubicin hydrochloride was purchased from Apollo Scientific Ltd. (Cheshire, UK). Fetal calf serum (FCS) was purchased from Biowest (Riverside, MO, USA). Dulbecco’s modified Eagle’s medium (DMEM) was purchased from Nissui Pharmaceutical (Tokyo, Japan).

### 2.2. Preparation of Hollow Nanocapsules 

Self-assembled PAMAM dendron-PLL polymer vesicles were prepared according to a previous report [18,19,20]. Briefly, PAMAM dendron-PLL was dissolved in a 50:50 (*v*/*v*) mixture of distilled water and methanol, and MeOH was then added dropwise with vigorous stirring to produce an 80 vol % methanol solution. The formation of PAMAM dendron-PLL polymer vesicles with a narrow size distribution was confirmed by dynamic light scattering (DLS). DLS measurements were carried out at 25 °C using an ELS-8000 (Otsuka Electronics Co., Ltd., Osaka, Japan) equipped with a He/Ne ion laser (λ = 633 nm). The DLS measurements utilized a 90° detection angle, and the average diameter was calculated by the Stokes–Einstein equation. Transmission electron microscopy (TEM) observation was carried out using a JEOL2000 (JEOL, Tokyo, Japan) instrument with carbon-coated copper grids; the sample was negatively stained with phosphotungstic acid. For the introduction of SH groups to PLL tails, 2-iminothiolane (IT) in H_2_O/MeOH at 80 vol % MeOH was added to a solution of the PAMAM dendron-PLL polymer vesicles, in which the molar ratio of IT and Lys residues was 0.25. After 1 day of the reaction, the unreacted IT was removed by dialysis against distilled water, and SS-bonded PAMAM dendron-PLL nanocapsules were obtained. Additionally, to obtain X-linked nanocapsules, EGDE instead of IT was added to a solution of the PAMAM dendron-PLL polymer vesicles, in which the molar ratio of EGDE and Lys residues was 1:1. The solvent exchange and removal of unreacted EGDE were performed by the same method used for the SS-bonded nanocapsules.

### 2.3. DOX Loading into Nanocapsules 

DOX loading into the nanocapsules was performed by mixing a nanocapsules solution with DOX in H_2_O/MeOH at 80 vol % MeOH. 2.5 mg of DOX was dissolved into 2.0 mL of nanocapsule solutions (2.5 mg/mL) and the solutions were mixed and stored overnight under dark conditions. The un-loaded DOX was removed by dialysis against phosphate buffered saline (PBS). The DOX concentration in the nanocapsules was determined from the absorbance at 485 nm, and the loading content (LC) and loading efficiency (LE) of DOX were calculated from the following equations:

$$\mathrm{Loading}\;\mathrm{content}\;\left(\%\right)=\;\frac{\mathrm{amount}\;\mathrm{of}\;\mathrm{DOX}\;\mathrm{in}\;\mathrm{nanocapsule}}{\mathrm{weight}\;\mathrm{of}\;\mathrm{nanocapsules}}\;\times \;100$$

$$\mathrm{Loading}\;\mathrm{efficiency}\;\left(\%\right)=\;\frac{\mathrm{amount}\;\mathrm{of}\;\mathrm{DOX}\;\mathrm{in}\;\mathrm{nanocapsule}}{\mathrm{loaded}\;\mathrm{amount}\;\mathrm{of}\;\mathrm{DOX}}\;\times \;100.$$

### 2.4. DOX Release from Nanocapsules

The release of DOX molecules from nanocapsules was evaluated by the dialysis method. Samples of 3.0 mL were placed into a dialysis cassette (Slide-A-Lyzer Dialysis Cassette, Thermo Scientific, Rockford, IL, USA) with MWCO 10,000, and the dialysis cassettes were immersed in 1.0 L of PBS including 10 μM of GSH at 37 °C. The concentration of GSH in the outer medium was increased to 0.5 mM after 24 h. One hundred microliter samples were collected from the dialysis cassette at predetermined times, and the absorbance at 485 nm was measured using a V-550 UV/Vis spectrophotometer (JASCO, Tokyo, Japan). The remaining amount of DOX in the dialysis cassettes was calculated using calibration curves of DOX in PBS.

### 2.5. Experiments Using Cultured Cells

To evaluate cellular uptake, intracellular distribution and cell viability of DOX-loaded nanocapsules, cell culturing was performed using HeLa cells. Cellular uptake was evaluated by using fluorescein isothiocyanate (FITC)-labeled nanocapsules, in which PAMAM dendron-PLL and FITC were reacted in a 50 mM borate buffer (pH 8.5), and unreacted FITC was removed by dialysis against distilled water. HeLa cells were seeded in 0.5 mL of DMEM with 10% FCS in 24-well culture plates at 5 × 10^4^ cells per well the day before the uptake experiments. The cells were washed with PBS and then covered with DMEM (1.0 mL). DOX-loaded nanocapsule solutions were gently added to the cells and incubated at 37 °C for varying incubation times (1, 2, 4, and 8 h). Cells were washed with PBS and detached using trypsin. Cellular fluorescence was then evaluated by flow cytometry (EPICS XL, Beckman Coulter, Inc., Brea, CA, USA). Intracellular distribution of DOX molecules was observed by confocal laser scanning microscopy using a LSM 5 EXCITER (Carl Zeiss Co. Ltd., Oberkochen, Germany). The cell viability of HeLa cells treated by DOX-loaded nanocapsules was evaluated by the MTT assay. HeLa cells were seeded in 100 μL of DMEM with 10% FCS in each well of 96-well plates at 1 × 10^4^ cells for 1 day. HeLa cells were incubated with the samples for 4 h. The culture medium was exchanged, and the cells were incubated for varying times (2, 8, 20, 28, and 44 h) in 100 μL of DMEM with 10% FCS. The culture medium was then replaced with DMEM with 10% FCS containing MTT. After 3 h of incubation, the medium was removed and the cells were solubilized in 2-propanol containing 0.1 M HCl. The viable cells were counted from the absorbance at 490 nm using an ARVO_SX_ multilabel counter (Perkin Elmer, Turku, Finland).

## 3. Results and Discussion

**Figure 1:**
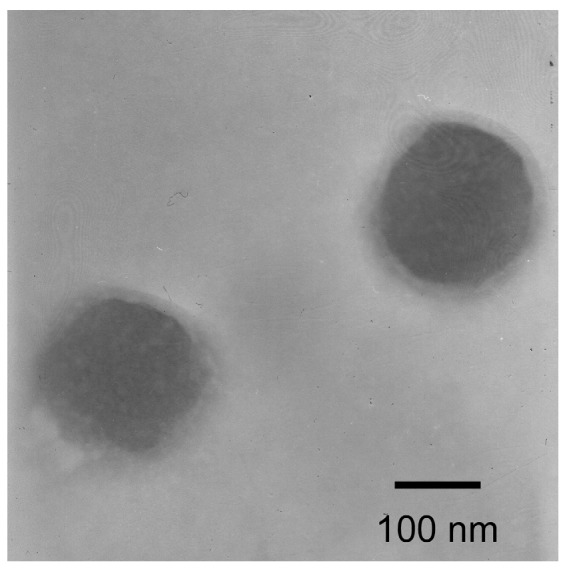
TEM image of hollow nanocapsules prepared though the stabilization of PAMAM dendron-PLL self-assembled polymer vesicles by introducing disulfide bonds.

**Figure 2:**
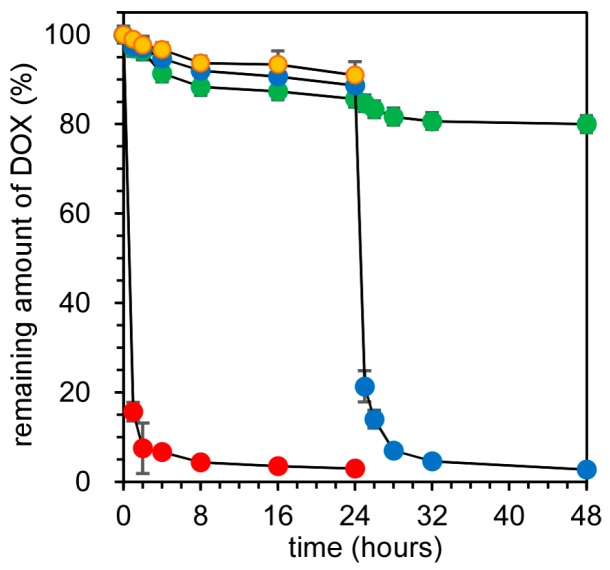
Release profiles of DOX from disulfide (SS)-bonded and X-linked nanocapsules at the extracellular GSH concentration (first 24 h incubation period, 10 μM) and the intracellular GSH concentration (second 24 h incubation period, 0.5 mM). Free DOX at pH 7.4, ●; DOX in SS-bonded nanocapsules at pH 7.4, ●; DOX in SS-bonded nanocapsules at pH 5.5, ●; DOX in X-linked nanocapsules at pH 7.4, ●. The data are the average of three experiments ± SD.

Two types of hollow nanocapsules, SS-bonded and X-linked nanocapsules, were prepared by stabilizing PAMAM dendron-PLL self-assembled polymer vesicles using an IT or EGDE in a MeOH/H_2_O mixture (80 vol % MeOH). Hollow nanocapsules were observed in TEM observation (Figure 1). DOX was loaded into the nanocapsules by mixing in MeOH/H_2_O (80 vol % MeOH). The LC and LE of the DOX, mean diameter, and polydispersity index (PDI) of DOX-loaded nanocapsules before and after DOX loading are summarized in Table 1. There was no significant difference in LC and LE between SS-bonded and X-linked nanocapsules, and their LC and LE were ca. 4% and ca. 8%, respectively. Additionally, the mean diameter and PDI of the nanocapsules were not influenced by DOX loading, and both nanocapsules had a mean diameter of ca. 200 nm with a narrow distribution (less than 0.1 of PDI). For confirming the effect of the electrostatic barrier of the nanocapsules against the leakage of loaded DOX, the release of DOX from SS-bonded and X-linked nanocapsules was evaluated by the dialysis method. In this experiment, GSH concentrations of the outer medium were increased at 24 h incubation from 10 μM to 0.5 mM, which correspond to extra- and intra-cellular GSH concentrations, respectively [23,24,25]. Figure 2 show the release profiles of DOX from SS-bonded and X-linked nanocapsules. At the extracellular GSH concentration (10 μM), the release rates of DOX from both SS-bonded and X-linked nanocapsules were very slow, and ca. 90% of DOX remained in the nanocapsules after 24 h of incubation. Additionally, DOX from SS-bonded nanocapsules was observed at endosomal pH (pH 5.5). Such a slow release is due to the effect of the electrostatic barrier of the cationic PLL membrane. We have previously compared the release of cationic and anionic fluorescence dyes (rhodamine 6G and fluorescein) from SS-bonded and X-linked nanocapsules. Although anionic fluorescein molecules are gradually released, cationic rhodamine 6G showed almost no release from the nanocapsules because of the electrostatic SS-bonded PLL membrane in the nanocapsules, in which cationic rhodamine 6G could not distribute into the cationic PLL membrane and diffuse into the outer medium. DOX has a primary amine with a p*K*_a_ 8.3 [21,22] and the protonated DOX/non-protonated DOX ratio is theoretically 8:1 at pH 7.4. Due to the cationic nature of DOX at pH 7.4, the DOX release from nanocapsules was very slow, as shown in Figure 2. When the GSH concentration of the outer medium increased to 0.5 mM after 24 h of incubation, DOX molecules were immediately released only from SS-bonded nanocapsules. The release rate of DOX in SS-bonded nanocapsules after 24 h of incubation and in the presence of 0.5 mM GSH is comparable with that of free DOX, suggesting that DOX molecules are released readily from nanocapsules in response to a high GSH concentration. On the other hand, the increase in GSH concentration in the outer medium had no influence to DOX release from X-linked nanocapsules. There was a critical GSH concentration for the collapse of SS-bonded nanocapsules between 20 and 50 μM [20]. By increasing the GSH concentration to an intracellular level, which is over the critical GSH concentration, SS-bonded nanocapsules were destabilized through dissociation of the SS bonds between PLL tails, and then, DOX release from the nanocapsules was immediate. Thus, SS-bonded nanocapsules can effectively release DOX molecules under the reductive conditions of the cytoplasm.

**Figure 3:**
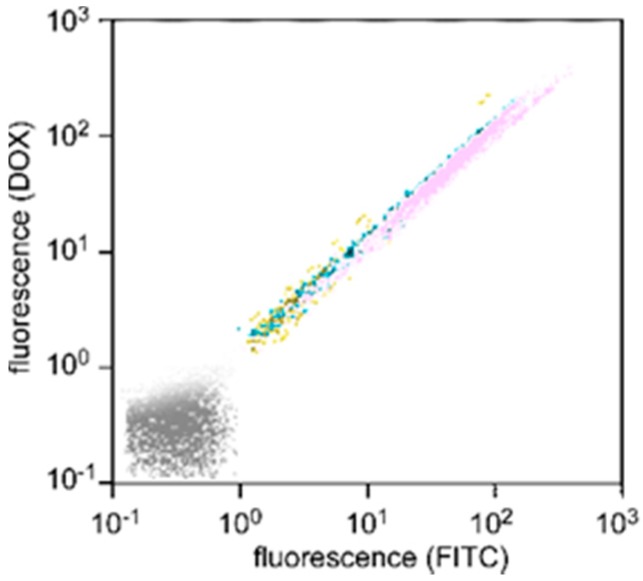
Time dependence of cellular uptake of DOX-loaded SS-bonded nanocapsules that were covalently labeled with fluorescein isothiocyanate (FITC). Control (without treatment): gray points; 1 h: yellow points; 2 h: green points; 4 h: blue points; 8 h: pink points.

The cellular uptake of DOX-loaded SS-bonded nanocapsules was evaluated using flow cytometry. Figure 3 shows the fluorescence distribution of HeLa cells treated with DOX-loaded SS-bonded nanocapsules for varying incubation periods, in which PAMAM dendron-PLL, i.e., nanocapsules, was covalently labeled with FITC. The fluorescence distribution of HeLa cells gradually shifted to a strong fluorescence intensity with a prolongation of incubation time for both red (DOX) and green (nanocapsule) fluorescence. Importantly, all plots showed distributions in accordance with the same straight line. This demonstrates that the uptake of DOX and nanocapsules by all cells was at the same ratio and independent of the incubation time, i.e., the nanocapsules did not release DOX before cellular uptake.

**Figure 4:**
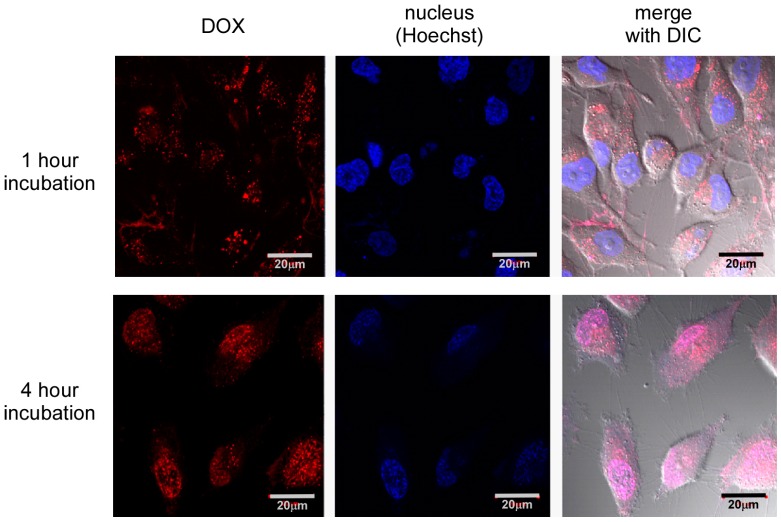
Intracellular distribution of DOX molecules loaded into SS-bonded nanocapsules after 1 h and 4 h incubation periods.

Further, intracellular distribution of DOX was confirmed by laser confocal microscopic observation (Figure 4). There was a significant difference in the intracellular distribution of DOX between 1 and 4 h incubation time. After 1 h of incubation, the DOX fluorescence did not distribute into the nucleus, although red fluorescence dots were observed in the cells. Since free DOX molecules can distribute into the nucleus spontaneously [26,27], this suggests that DOX molecules were not release from nanocapsules, and DOX-loaded nanocapsules were mainly located in endosomes. In contrast, DOX red fluorescence overlapped with Hoechst blue fluorescence after 4 h of incubation, demonstrating DOX molecules were distributed in the nucleus. This change in intracellular distribution of DOX between 1 and 4 h of incubation indicates successful delivery of DOX into the cytoplasm. In this time period, DOX-loaded SS-bonded nanocapsules can escape from the endosome by the buffering effect of tertiary amines in the interior of the PAMAM dendron, and DOX molecules are released through the collapse of nanocapsules by the cleavage of SS bonds in response to the high GSH concentration. That is, these results indicate that pH and redox dual responsibilities of SS-bonded nanocapsules can work in cultured cells.

**Figure 5:**
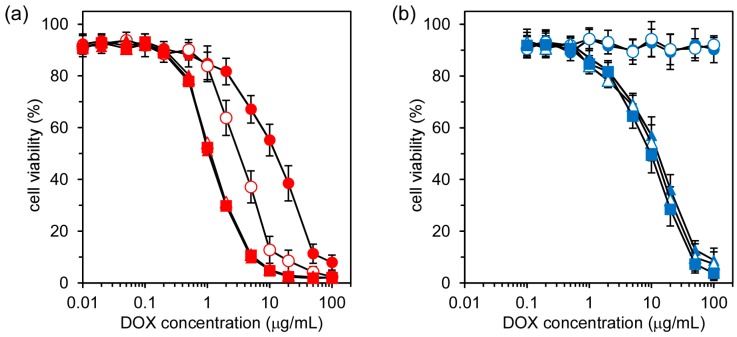

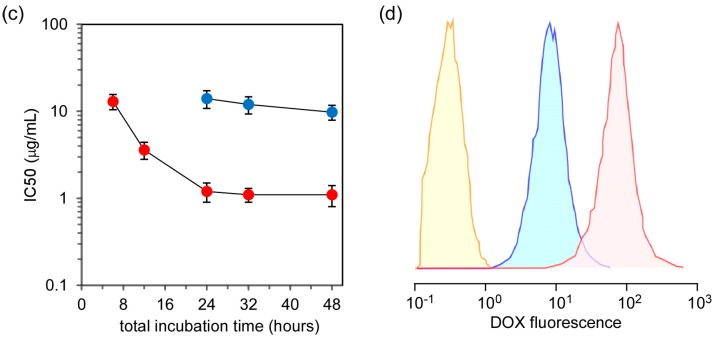
Anticancer effect of free DOX and DOX in SS-bonded nanocapsules to HeLa cells. The change in cell viabilities with different DOX concentrations and incubation periods for free DOX (**a**) and DOX in SS-bonded nanocapsules (**b**); Incubation time dependence of IC50 values for free DOX and DOX in SS-bonded nanocapsules (**c**); Fluorescence distribution of HeLa cells treated with free DOX and DOX in SS-bonded nanocapsules for 4 h (**d**). In (**a**,**b**), the plots for 6, 12, 24, 32, and 48 h total incubation are represented by closed circles, open circles, closed triangles, open triangles, and closed squares, respectively. The data are the average of three experiments ± SD. In (**c**), free DOX and DOX in SS-bonded nanocapsules are represented by red and blue plots, respectively. In (**d**), free DOX, DOX in SS-bonded nanocapsules and control (without treatment) are represented by red, blue, and yellow distributions.

Finally, the in vitro anticancer effect of DOX in nanocapsules to HeLa cells was evaluated by the MTT assay. Here, DOX entrapped in nanocapsules was incubated with HeLa cells for 4 h, and the MTT assay was performed after different incubation periods. Figure 5a,b show the cell viabilities of HeLa cells treated by free DOX and DOX in SS-bonded nanocapsules at different total incubation periods. For free DOX, the cell viability decreased steeply at certain DOX concentrations, and this cell viability decrease at particular DOX concentrations was also dependent on the incubation time, with longer incubation periods leading to greater cell viability loss (Figure 5a). In the case of DOX in SS-bonded nanocapsules, there was no reduction in cell viability after 6 and 12 h incubation periods. However, steep decreases in cell viability were observed for incubation periods of 24, 32, and 48 h (Figure 5b). There are two possibilities for this decrease in cell viability: one is an anticancer effect of DOX and the cytotoxicity of nanocapsules. There was no decrease in the viability of the cells treated by the empty SS-bonded nanocapsules, in which the concentration of the empty nanocapsules was 1.2 mg/mL, corresponding to the highest DOX concentration (100 μg/mL) in Figure 5b. Their cell viabilities were maintained at more than 85% under the same experimental conditions. This indicates that nanocapsules had negligible cytotoxicity and the decrease in cell viability in Figure 5b was due to the anticancer effect of DOX. Figure 5c shows the change in IC50 (50% inhibitory concentration) values, which were determined from Figure 5a,b, for free DOX and DOX in SS-bonded nanocapsules at varying total incubation periods. The delay of the anticancer effect of DOX in SS-bonded nanocapsules compared with free DOX is due to the difference in the cellular uptake pathway. DOX molecules can penetrate into the cytoplasm and accumulate in the nucleus. On the other hand, DOX in SS-bonded nanocapsules are taken up into the cells via the endocytosis pathway as shown in Figure 3 and the accumulation in the nucleus of DOX in SS-bonded nanocapsules takes longer when compared with the uptake of free DOX. Additionally, even after a 48 h incubation period, the IC50 value of DOX in SS-bonded nanocapsules is 8.9 times higher than that of free DOX. This low anticancer effect of DOX in SS-bonded nanocapsules is due to the difference of DOX uptake by the cells. Figure 5d shows the fluorescence distribution of HeLa cells treated with free DOX and DOX in SS-bonded nanocapsules after 4 h of incubation, which is the incubation time of samples with the cells. Clearly, the amount of DOX taken up by the cells treated with free DOX is high when compared with that of DOX in SS-bonded nanocapsules, and the mean fluorescence intensity of HeLa cells treated with free DOX is 11.7 times greater than the value obtained for cells treated by DOX in SS-bonded nanocapsules. Additionally, the mean fluorescence intensity of HeLa cells did not increase with a prolongation of DOX treatment time, suggesting that all DOX molecules were taken up into the cells for 4 h of treatment. The percentage of the nanocapsules taken up was calculated to be 8.5% based on the assumption that all DOX molecules were taken up into the cells at 4 h. Furthermore, considering the difference in IC50 values and the amount of DOX uptake by cells between free DOX and DOX in SS-bonded nanocapsules, DOX in nanocapsules exhibits a comparable or more effective anticancer effect than free DOX. This suggests the smooth release of DOX molecules from SS-bonded nanocapsules through the collapse of the nanocapsules by SS-bond cleavage in response to the reductive environment in the cytoplasm.

## 4. Conclusions

The pH and redox dual responsive hollow nanocapsules were prepared through the stabilization of PAMAM dendron-PLL self-assembled polymer vesicles by introducing SS bond linkages between PLLs, in which tertiary amines in the interior of the PAMAM dendron exhibit a buffering effect for regulating endosomal escape. SS-bonded nanocapsules effectively inhibit the release of DOX because of the electrostatic barrier of the SS-bonded PLL membrane. This SS-bonded PLL membrane is stable under the extracellular GSH concentration and 90% DOX molecules are maintained in the nanocapsules even after 24 h of incubation. DOX molecules are released from nanocapsules in response to a rise in the intracellular GSH concentration, since SS bonds are cleaved at the intracellular GSH concentration. The PAMAM dendron also showed a buffering effect for endosomal escape, and SS-bonded nanocapsules delivered DOX molecules into the cytoplasm through a smooth release in response to the intracellular GSH concentration. The in vitro anticancer effect of DOX in SS-bonded nanocapsules was similar or more effective when compared with that of free DOX. Thus, the pH and redox dual responsive hollow nanocapsules, which were prepared from PAMAM dendron-PLL self-assembled polymer vesicles bearing an electrostatic barrier, are suitable for the delivery of cationic molecules into the cytoplasm.

## Figures and Tables

**Table 1:** Preparation of hollow nanocapsules for DOX delivery. *

Type of Nanocapsules	Mean Diameter (nm)/PDI **		LC (%)	LE (%)
	Before DOX Loading	After DOX Loading		
SS-bonded	196.1 ± 1.9/0.08 ± 0.01	193.6 ± 3.6/0.08 ± 0.01	4.1 ± 0.3	8.2 ± 0.5
X-linked	200.0 ± 7.2/0.09 ± 0.02	198.3 ± 4.5/0.09 ± 0.01	3.9 ± 0.4	7.9 ± 0.7

* The data are the average of three experiments ± SD. ** These values were determined from the cumulant analysis of DLS.
